# Supplementary material for: Mitochondrial phylogeny and comparative mitogenomics of closely related pine moth pests (Lepidoptera: Dendrolimus)
Source: PeerJ. 2019 Jul 23;7:e7317. doi: 10.7717/peerj.7317 (PMC6659665; doi:10.7717/peerj.7317)
Supplement: Supplemental Information 2 — (A) Phylogenetic tree (ML) of lepidoptera (moth) with outgroups constructed with nucleotides sequence of 13 protein coding genes. (B) Phylogenetic tree (BI) of lepidoptera (moth) with outgroups constructed with nucleotides sequence of 37 genes. (C) Phylogenetic tree (ML) of lepidoptera (moth) with outgroups constructed with nucleotides sequence of 37 genes. Clades with different colors indicate different superfamily. The names of superfamilies were labelled on the tree. Numbers above or below branches indicate bootstrap value. [file peerj-07-7317-s002.docx]

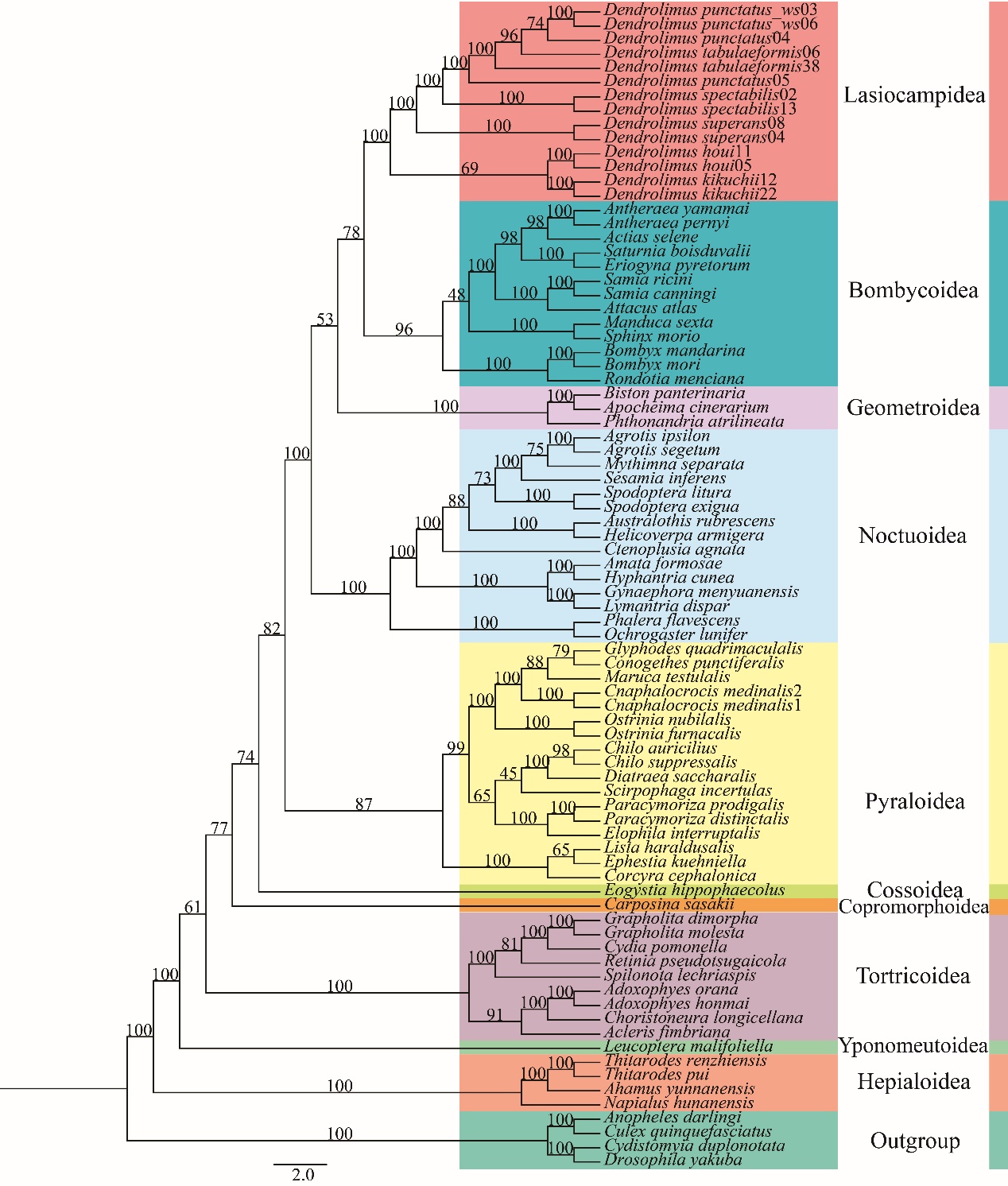


Supplemental Information 2 (A) Phylogenetic tree (ML) of Lepidoptera (moth) with outgroups constructed with nucleotides sequence of 13 protein coding genes. Numbers above or below branches indicate bootstrap value. Clades with different colors indicate different superfamily. The names of superfamilies were labelled on the tree.


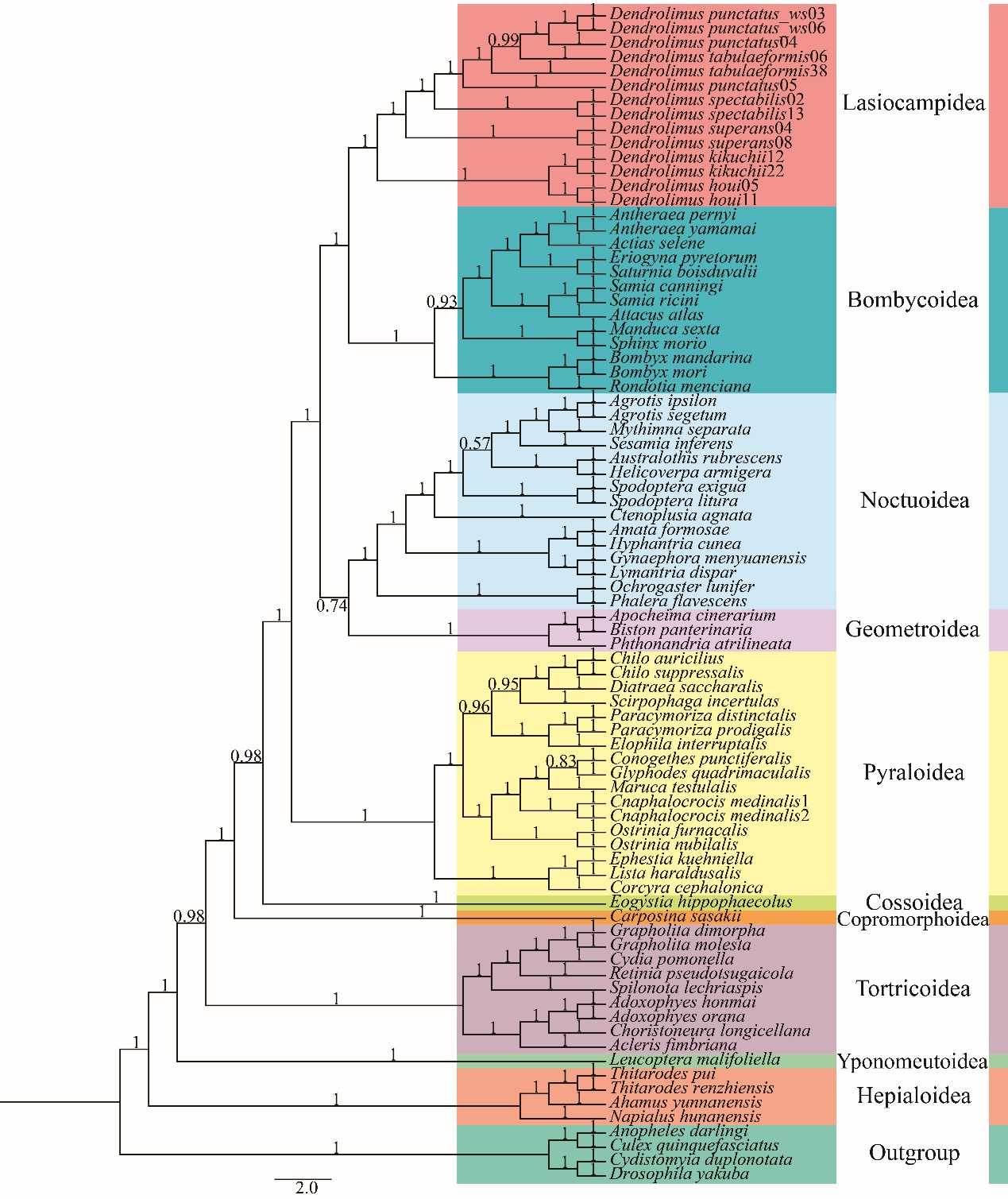


Supplemental Information 2 (B) Phylogenetic tree (BI) of Lepidoptera (moth) with outgroups constructed with nucleotides sequence of 37 genes. Numbers above or below branches indicate posterior probabilities. Clades with different colors indicate different superfamily. The names of superfamilies were labelled on the tree.


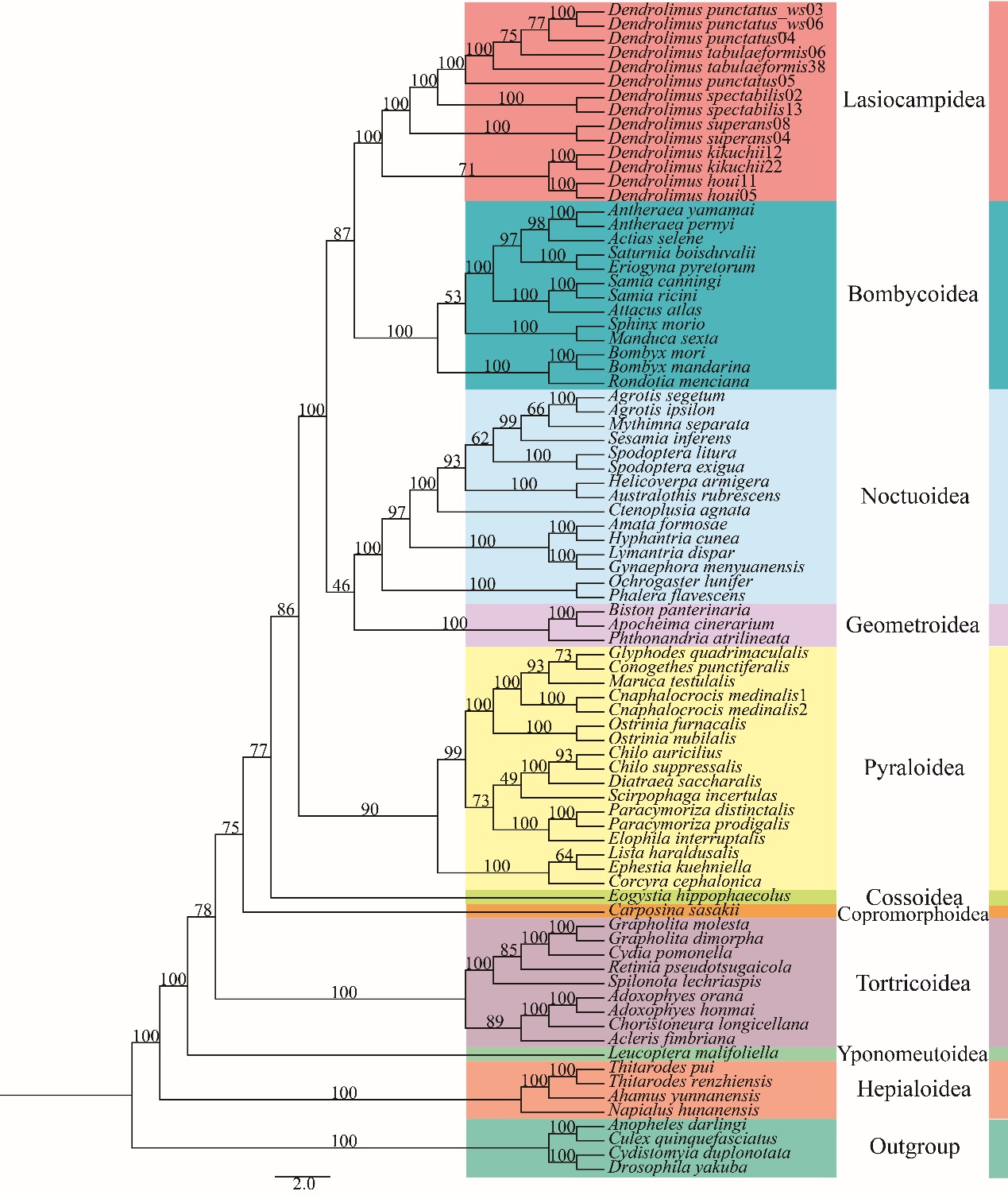


Supplemental Information 2 (C) Phylogenetic tree (ML) of Lepidoptera (moth) with outgroups constructed with nucleotides sequence of 37 genes. Numbers above or below branches indicate bootstrap values. Clades with different colors indicate different superfamily. The names of superfamilies were labelled on the tree.
