# Supplementary material for: Mitochondrial phylogeny and comparative mitogenomics of closely related pine moth pests (Lepidoptera: Dendrolimus)
Source: PeerJ. 2019 Jul 23;7:e7317. doi: 10.7717/peerj.7317 (PMC6659665; doi:10.7717/peerj.7317)
Supplement: Supplemental Information 4 — Codon Families are provided on the x axis. Codons that are absent in the mitochondrial genomes are marked at the top of columns Leul stands for Leu (CUN), Leu2 stands for Leu (UUR), Serl stands for Ser (AGN), Ser2 stands for Ser (UCN) [file peerj-07-7317-s004.docx]

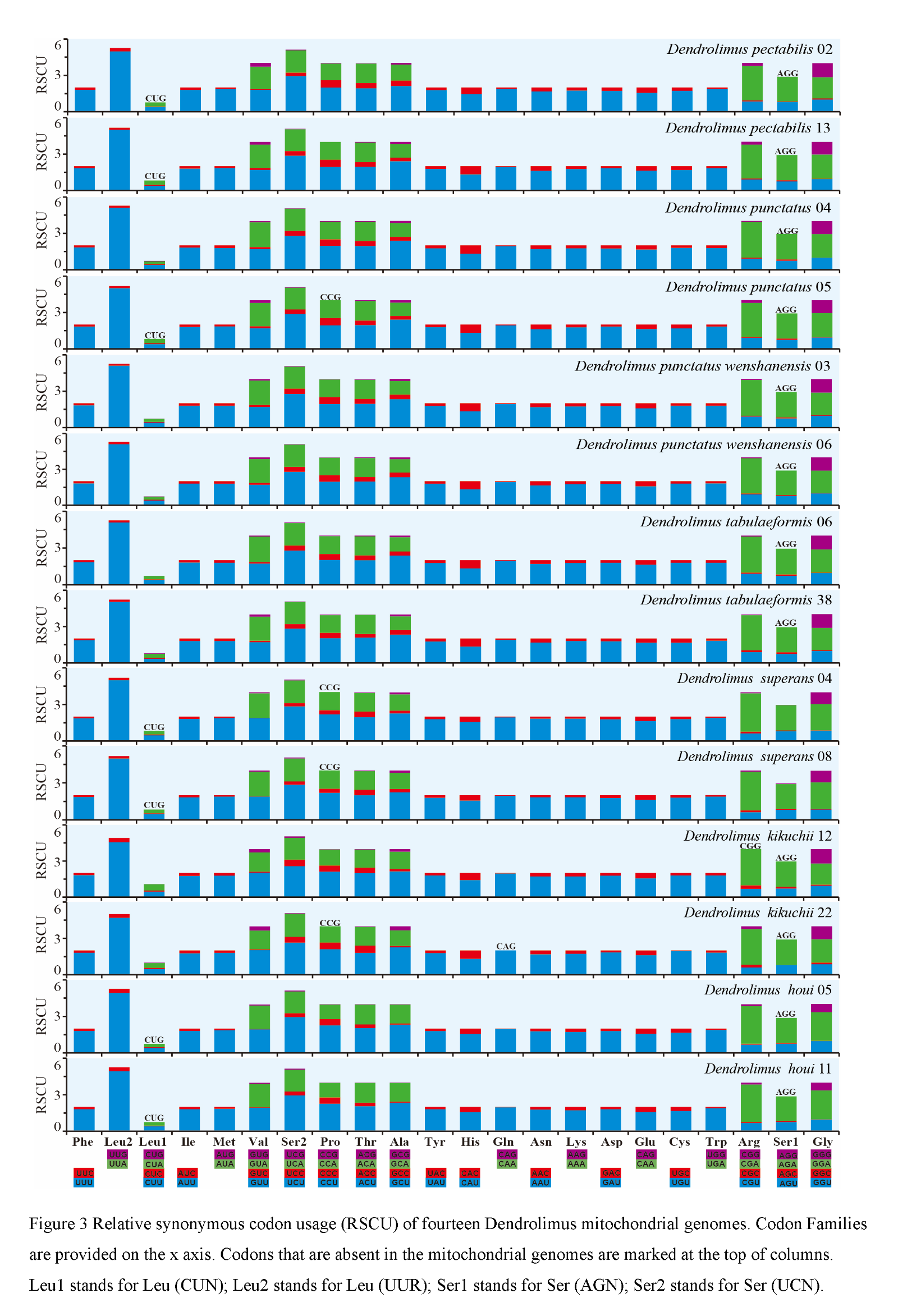


Supplemental Information 4 Relative synonymous codon usage (RSCU)of fourteen *Dendrolimus* mitochondrial genomes. Codon Families are provided on the x axis. Codons that are absent in the mitochondrial genomes are marked at the top of columns Leul stands for Leu(CUN), Leu2 stands for Leu(UUR), Serl stands for Ser(AGN), Ser2 stands for Ser(UCN)
