# Supplementary material for: Mitochondrial phylogeny and comparative mitogenomics of closely related pine moth pests (Lepidoptera: Dendrolimus)
Source: PeerJ. 2019 Jul 23;7:e7317. doi: 10.7717/peerj.7317 (PMC6659665; doi:10.7717/peerj.7317)
Supplement: Supplemental Information 5 — Numbers above the column refer to the number of codons. CDSPT stands for codons per thousand codons Codon Families are provided on the x axis. Leul stands for Leu(CUN): Leu stands for Leu(UUR), Serl stands for Ser(AGN), Ser2 stands for Ser(UCN). Additional file 5 (A) Phylogenetic tree (ML) of Dendrolimus species constructed with A+T rich region. (B) Phylogenetic tree (ML) of Dendrolimus species constructed with intergenic region. Numbers above or below branches indicate bootstrap value. [file peerj-07-7317-s005.docx]

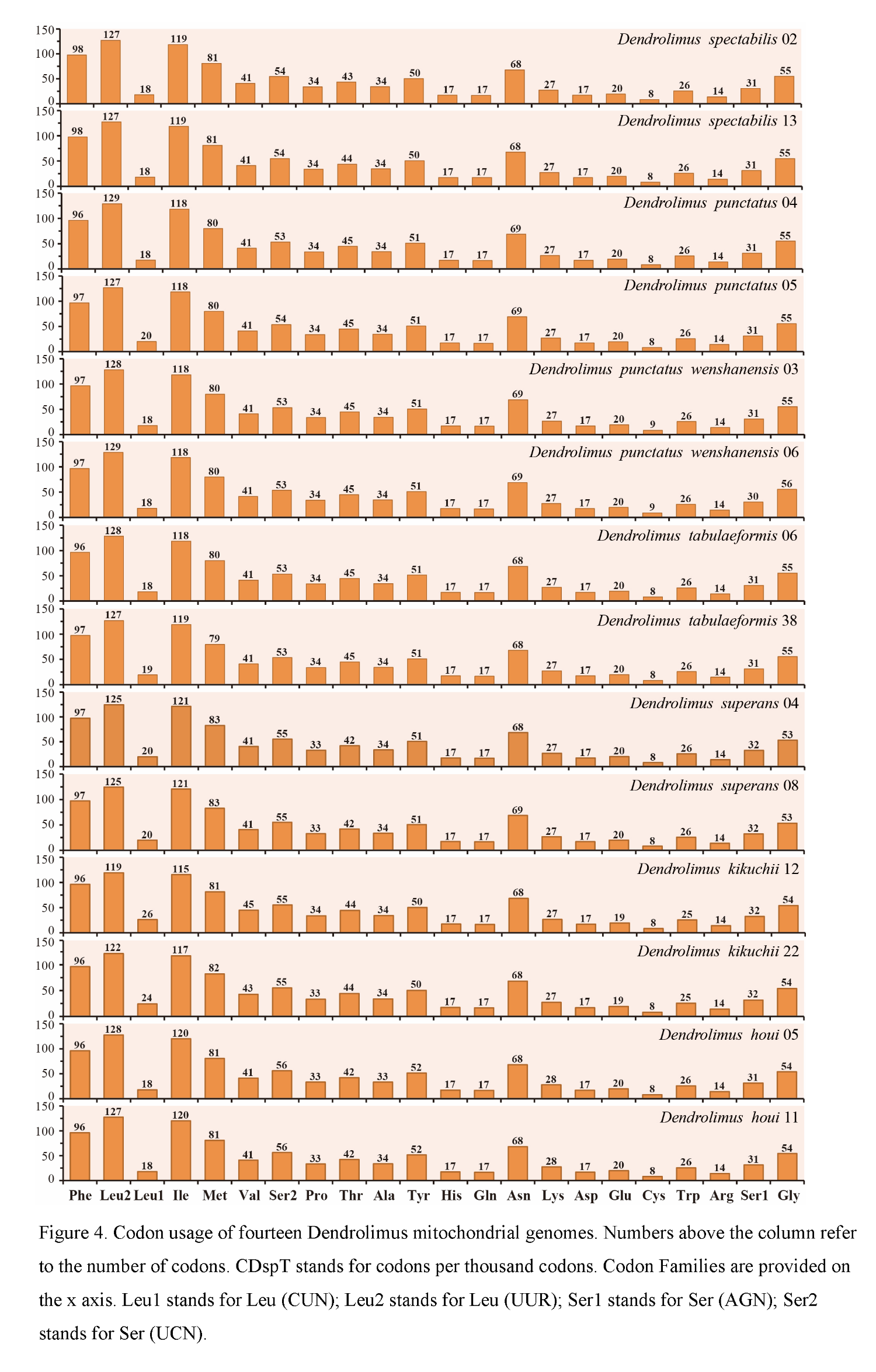

Supplemental Information 5 Codon usage of fourteen *Dendrolimus* mitochondrial genomes. Numbers above the column refer to the number of codons. CDSPT stands for codons per thousand codons Codon Families are provided on the x axis. Leul stands for Leu(CUN): Leu stands for Leu(UUR), Serl stands for Ser(AGN), Ser2 stands for Ser(UCN).
