# Supplementary material for: Mitochondrial phylogeny and comparative mitogenomics of closely related pine moth pests (Lepidoptera: Dendrolimus)
Source: PeerJ. 2019 Jul 23;7:e7317. doi: 10.7717/peerj.7317 (PMC6659665; doi:10.7717/peerj.7317)
Supplement: Supplemental Information 6 — (A) Phylogenetic tree (ML) of Dendrolimus species constructed with A+T rich region. (B) Phylogenetic tree (ML) of Dendrolimus species constructed with intergenic region. Numbers above or below branches indicate bootstrap value. [file peerj-07-7317-s006.docx]

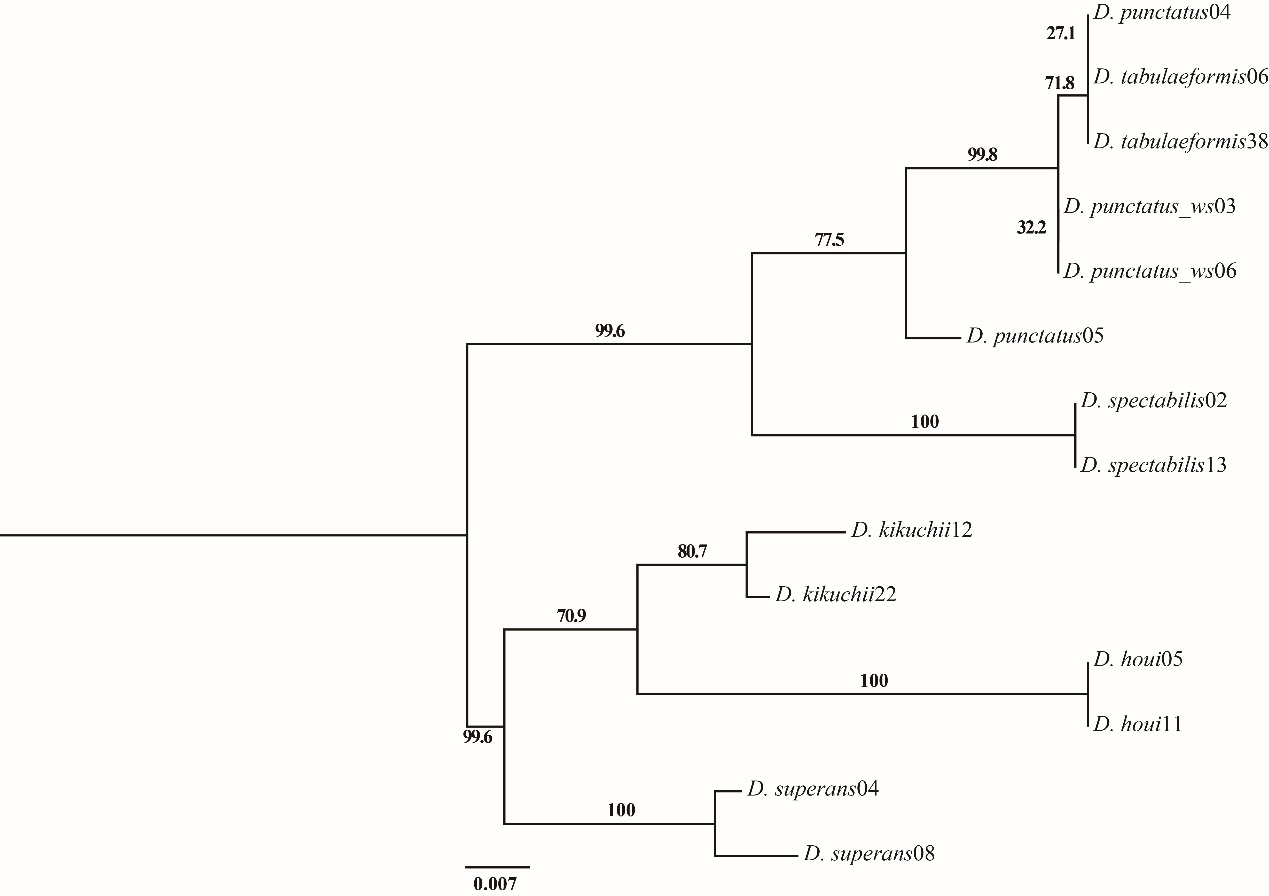


Supplemental Information 6 (A) Phylogenetic tree (ML) of *Dendrolimus* species constructed with A+T rich region. Numbers above or below branches indicate bootstrap value.


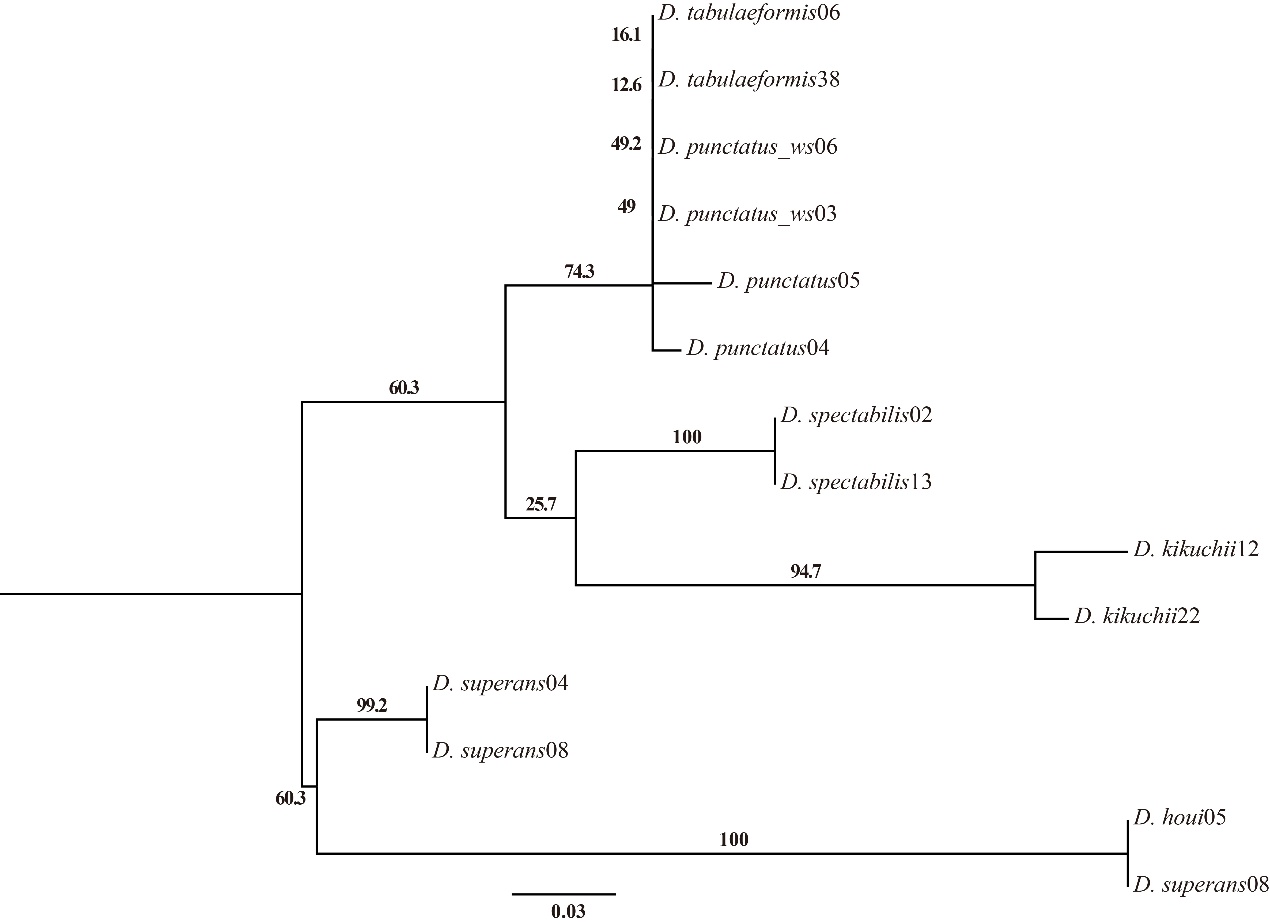


Supplemental Information 6 (B) Phylogenetic tree (ML) of *Dendrolimus* species constructed with intergenic region. Numbers above or below branches indicate bootstrap value.
